# The risk of dementia in adults with abdominal aortic aneurysm

**DOI:** 10.1038/s41598-022-05191-1

**Published:** 2022-01-24

**Authors:** Hyung-jin Cho, Ju-hwan Yoo, Mi-hyeong Kim, Kyung-jai Ko, Kang-woong Jun, Kyung-do Han, Jeong-kye Hwang

**Affiliations:** 1 Division of Vascular and Transplant Surgery, Department of Surgery, Eunpyeong St. Mary’s Hospital, College of Medicine, The Catholic University of Korea, 1021, Tongil-ro, Eunpyeong-gu, Seoul, 03312 Korea; 2 Department of Biomedicine and Health Science, The Catholic University of Korea, Seoul, Korea; 3 Department of Surgery, Kangdong Sacred Heart Hospital, Seoul, Korea; 4 Division of Vascular and Transplant Surgery, Department of Surgery, Bucheon St. Mary’s Hospital, College of Medicine, The Catholic University of Korea, Bucheon, Gyeonggi-do Korea; 5 Department of Statistics and Actuarial Science, Soongsil University, 369 Sangdo-ro, Dongjak-gu, Seoul, 06978 Korea

**Keywords:** Diseases, Neurology, Risk factors

## Abstract

Abdominal aortic aneurysm (AAA) and dementia have similar epidemiological profiles and common pathogenic mechanisms. However, there have been few studies on the link between these two diseases. For this study, information from 2009 to 2015 was extracted from the Korean National Health Insurance system database. A total of 15,251 participants with a new diagnosis of AAA was included. Propensity score matching by age and sex with patients in whom AAA was not diagnosed was used to select the control group of 45,753 participants. The primary endpoint of this study was newly diagnosed dementia (Alzheimer’s disease (AD), vascular dementia (VD), or other type of dementia). The incidence of dementia was 23.084 per 1000 person years in the AAA group, which was higher than that of the control group (15.438 per 1000 person years). When divided into AD and VD groups, the incidence of AD was higher than that of VD, but the HR of AAA for occurrence of dementia was higher in VD (1.382 vs. 1.784). Among the various risk factors, there was an interaction of age, hypertension, and history of cardiovascular disease with incidence of dementia (p < 0.05). In the presence of hypertension, the HR for occurrence of dementia was high according to presence or absence of AAA (1.474 vs 1.165). In addition, this study showed higher HR in the younger age group (age < 65) and in the group with no history of cardiovascular disease [1.659 vs. 1.403 (age), 1.521 vs. 1.255 (history of cardiovascular disease)]. AAA was associated with increased risk of dementia regardless of AD or VD, even after adjusting for several comorbidities. These findings indicate that follow-up with AAA patients is necessary for early detection of signs and symptoms of dementia.

## Introduction

Dementia is acquired loss of cognition in multiple cognitive domains that is sufficiently severe to affect social or occupational function^1^. The pooled point prevalence of dementia per 1000 in 23 community setting studies was 48.62, and the incidence rate of dementia per 1000 person-years was 17.18 in the community-only setting. The three most common types of dementia in order of prevalence are Alzheimer’s disease (AD), vascular dementia (VD), and Lewy body dementia^2^. Age is the most important risk factor for dementia, with prevalence doubling every 5 years after age 65; the prevalence is increased from 2–3% in those 65–69 years to 30% among individuals older than 80^3^.

An abdominal aortic aneurysm (AAA) is a permanent localized dilatation of the abdominal aorta that exceeds the normal diameter by 50%, or > 3 cm. The mean annual incidence of new AAA diagnoses in Western populations is 0.4–0.67%^4^. Age is a risk factor for AAA, and there are similarities in epidemiological profiles between dementia and AAA including smoking, hypertension, obesity, and hyperlipidemia^2,4,5^.

Until recently, dementia has been considered neither preventable nor treatable, but recent clinical trials assessing the effects of lifestyle modification in dementia offer hope, showing that 35% of AD risk is modifiable^2,6^. These modifiable factors generally do not apply to AAA; however, given the similarity of their epidemiological profiles and the many common pathogenic mechanisms^7^, the possibility of a link between the two can be considered.

Therefore, in this study, we investigated the effects of the presence of AAA on the incidence rate of dementia during a 7-year period in Korean using a Korean National Health Insurance Service (NHIS) database.

## Methods

### Data source

The Korean National Health Insurance (NHI) system consists of two major health care programs for universal coverage of all residents of Korea: NHI and Medical Aid (MA). Approximately 97% of the population is covered by NHI, and the remaining 3% is covered by MA^8^. The NHIS provides biannual health examinations for all insured Koreans and maintains an extensive data set of Koreans that includes patient demographics, medical treatment and procedures, and disease diagnoses according to the International Classification of Disease, 10th editions (ICD-10). For this study, information from 2009 to 2015 was extracted from the NHIS database.

### Sampled patients

**Figure 1:**
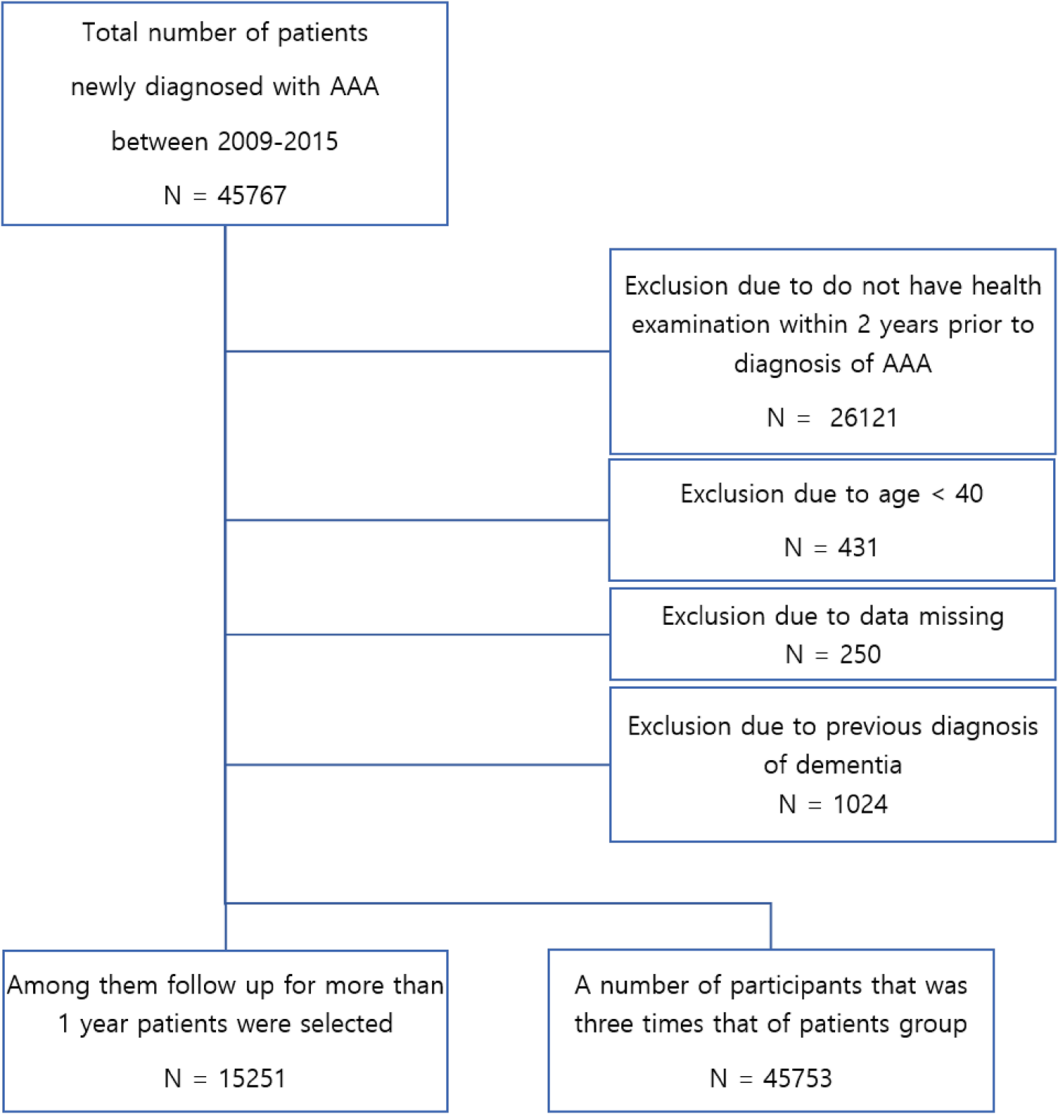
Enrollment flow chart.

Patients in the database who were newly diagnosed with AAA between 2009 and 2015 were enrolled (n = 45,767). The AAA group was defined as patients diagnosed with AAA codes (I71.3–I71.6, I71.8, and I71.9) more than twice at an outpatient department in the past year, who had been hospitalized with one of these AAA codes more than once, or who had undergone aneurysm repair surgery such as open surgical aneurysm repair (OSAR) or endovascular aneurysm repair (EVAR) defined by with the codes (O0223, O0224, O0234, M6611, and M6612). We excluded patients who had not undergone a health examination within 2 years prior to diagnosis of AAA (n = 26,121), were younger than 40 years (n = 431), had data missing (n = 250), or had a previous diagnosis of dementia (n = 1024). Among the remainder, a group of patients who were followed for more than one year was selected (n = 15,251). To select the control group, with a number of participants three times larger than that of the patient group (n = 45,753), we used propensity score matching by age and sex with patients in whom AAA was not diagnosed (Fig. 1). The primary endpoint of this study was newly diagnosed dementia (AD, VD, or other type of dementia).

### Data collection and definition

We collected baseline data from the NHIS database of age, sex, smoking status, alcohol consumption, physical activity, waist circumference, body mass index (BMI), and income level. Data about comorbidities, including hypertension, diabetes, dyslipidemia, chronic kidney disease (CKD), and history of cardiocerebrovascular disease (CVD), also were collected. Smoking status was classified as non-smoker, ex-smoker, and current smoker. Alcohol consumption was classified as heavy drinker when the average daily alcohol intake was 30 g or more, mild to moderate drinker when the individual consumed less than 30 g daily, and non-drinker. Physical activity was divided into two groups, one performing moderate exercise more than 5 days per week or vigorous exercise more than 3 days per week; and all others were classified into the second group. Income level was classified into a group that received medical aid and was in the bottom 20% of income and a group of all others. Comorbidities were defined as follows: DM [codes (E11-14 with antidiabetic medication) or (fasting blood sugar ≥ 126)], hypertension [codes (I10-13 and I-15 with antihypertensive medication) or (SBP ≥ 140) or (DBP ≥ 90)], dyslipidemia [(code E78 with antihyperlipidemic medications) or (total cholesterol ≥ 240)], CKD (eGFR < 60), and history of CVD [cerebrovascular disease codes (I60-64) and cardiovascular disease codes (I20-25)]. Finally, dementia was defined as follows; AD [codes (F00, and G30)], VD [code (F01)], other dementia [codes (F02, F03, and G31)] with one or more medications for dementia (donepezil, rivastigmine, galantamine, or memantine). When there were codes for both AD and VD, the main diagnosis was selected as the final diagnosis. If there was neither AD nor VD as a main diagnosis up to the second claim database, the diagnosis was defined as “other dementia”. This study is approved by the Institutional Review Board of The Catholic University of Korea, Eunpyeong St. Mary’s Hospital, Seoul, Korea (approval no. PC20ZISI0145). All methods were performed in accordance with the relevant guidelines and regulations approved by the Institutional Review Board of The Catholic University of Korea, Eunpyeong St. Mary’s Hospital, Seoul, Korea. Also, Informed consent was waived by the Institutional Review Board of the Catholic University of Korea, Eunpyeong St. Mary’s Hospital, Seoul, Korea because all analysis used anonymous data.

### Statistical analysis

Continuous variables are presented as mean ± standard deviation, and categorical variables are presented as number (percentage). To compare characteristics between patient and control groups, Student’s t-test was used for continuous variables and the chi-square test or Fisher’s exact test for categorical variables. The incidence rates of dementia are presented per 1000 person-years. Multivariate Cox regression models were used to evaluate the association of absence or presence of AAA with incidence of new-onset dementia. Model 1 examined the unadjusted hazard ratios (HRs). Model 2 was adjusted for age, sex, income level, presence of diabetes, hypertension, and dyslipidemia. In Model 3, smoking status, alcohol consumption, exercise status, and BMI were added; and history of CVD was added to Model 4. The cumulative incidence of dementia according to presence of AAA was calculated using Kaplan–Meier curves; the log-rank test was performed to analyze differences among the groups. Statistical significance was set at p < 0.05. All statistical analyses were performed using SAS version 9.4 (SAS Institute Inc., Cary, NC, USA.) and the R Project for Statistical Computing version 3.3.

## Results

### Baseline characteristics according to presence of AAA

The characteristics of subjects by presence of AAA are presented in Table 1. Hypertension, and dyslipidemia are known risk factors for AAA and were more common in the AAA group. Also, there were more smokers in the AAA group, and BMI and abdominal circumference were higher in the AAA group. In addition, history of CVD was significantly higher in the AAA group, but alcohol consumption and exercise level were lower in the AAA group.

**Table 1:** Clinical characteristics of control and abdominal aortic aneurysm (AAA) patients.

Variables	AAA		p-value
	No	Yes	
	45,753	15,251	
Age	66.08 ± 10.14	66.08 ± 10.14	1
Sex, male	29,874(65.29)	9958(65.29)	1
**Smoking status**			** < .0001**
Non	26,776(58.52)	7641(50.1)	
Ex	10,772(23.54)	3666(24.04)	
Current	8205(17.93)	3944(25.86)	
**Alcohol consumption**			** < .0001**
None	28,530(62.36)	10,207(66.93)	
Mild to moderate	14,181(30.99)	4087(26.8)	
Heavy	3042(6.65)	957(6.27)	
Regular exercise	10,473(22.89)	3147(20.63)	** < .0001**
Diabetes mellitus	9059(19.8)	3196(20.96)	**0.002**
Hypertension	24,564(53.69)	12,072(79.16)	** < .0001**
Dyslipidemia	15,050(32.89)	8827(57.88)	** < .0001**
Income level, Low	8683(18.98)	2799(18.35)	0.0872
BMI	23.93 ± 3.04	24.05 ± 3.15	** < .0001**
Waist circumference	83.19 ± 8.48	84.23 ± 8.78	** < .0001**
SBP	127.45 ± 15.41	128.52 ± 16.49	** < .0001**
DBP	77.37 ± 9.89	78.34 ± 10.72	** < .0001**
Glucose	104.26 ± 27.5	102.33 ± 25.39	** < .0001**
Cholesterol	193.03 ± 40.19	192.93 ± 43.44	0.7955
History of cerebrovascular disease	2512(5.49)	2348(15.4)	** < .0001**
History of cardiovascular disease	5653(12.36)	7762(50.9)	** < .0001**

*BMI* Body mass index, *SBP* Systolic blood pressure, *DBP* Diastolic blood pressure.

### Incidence of dementia

The incidence of dementia was 23.084 per 1000 person years in the AAA group, which was higher than that of the control group. When all models were applied respectively, the incidence rate remained significantly higher in the AAA group; and the hazard ratio was 1.516, 1.625, 1.568, and 1.422 for models 1 through 4, respectively. When divided into AD and VD groups, the incidence of AD was higher than that of VD, but the HR of AAA for dementia was higher in the VD group (1.382 vs. 1.784 in model 4). (Table 2).

**Table 2:** Hazard ratio of AAA for incidence of dementia.

AAA	N	Event	Duration	Rate	Model 1	Model 2	Model 3	Model 4
**All types of dementia**								
No	45,753	3234	209,489.14	15.4376	1	1	1	1
Yes	15,251	1432	62,034.29	23.084	1.516(1.424,1.613)	1.625(1.523,1.734)	1.568(1.468,1.674)	1.422(1.326,1.525)
**Alzheimer’s disease**								
No	45,753	2611	209,489.14	12.4637	1	1	1	1
Yes	15,251	1090	62,034.29	17.5709	1.433(1.335,1.538)	1.548(1.438,1.666)	1.495(1.388,1.61)	1.382(1.277,1.496)
**Vascular dementia**								
No	45,753	332	209,489.14	1.58481	1	1	1	1
Yes	15,251	213	62,034.29	3.43358	2.18(1.835,2.59)	2.233(1.865,2.672)	2.136(1.781,2.561)	1.784(1.467,2.169)

*AAA* Abdominal aortic aneurysm.

*Model 1* adjusted for age and sex, *Model 2* adjusted for age, sex, income level, diabetes, hypertension, and dyslipidemia. *Model 3* adjusted for age, sex, income level, diabetes, hypertension, dyslipidemia, smoking status, alcohol consumption, exercise status, and BMI. *Model 4* adjusted for age, sex, income level, diabetes, hypertension, dyslipidemia, smoking status, alcohol consumption, exercise status, BMI, and history of CVD.

**Figure 2:**
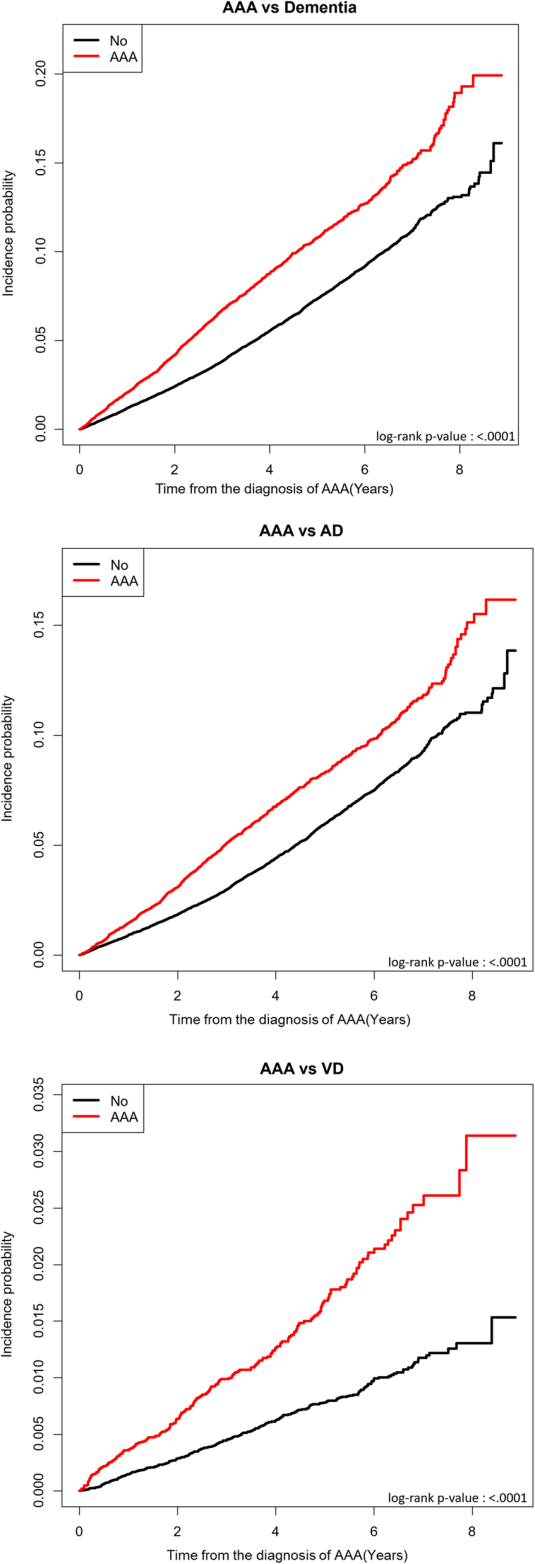
Kaplan–Meier plot for incidence of dementia in patients with AAA.

The Kaplan–Meier plot shows a comparison with the control group. All types of dementia occurred more frequently in the AAA group, which also was observed when divided into AD and VD groups (log rank test p value < 0.001) (Fig. 2).

### Interactions with AAA on occurrence of dementia

Among the factors, there was interaction of age, hypertension, and history of cardiovascular disease with AAA (p < 0.05). In the presence of hypertension, the HR for occurrence of dementia was high according to presence or absence of AAA (1.474 vs 1.165). These results were observed in the younger age group and in the group with no history of cardiovascular disease [1.659 vs. 1.403 (age), 1.521 vs. 1.255 (history of cardiovascular disease)] (Supplemental Table 1).

## Discussion

The current social expenditure of dementia is US$ 604 billion/year worldwide, and it is projected that the number of dementia patients will triple by 2050. Therefore, it is possible to estimate the global socio-economic impact of dementia^6,9^. For treatment of dementia, a variety of pharmacologic and non-pharmacologic approaches has been attempted, but their efficacy has not been confirmed. Rather, treatment is focused on symptom control, and prevention is considered the top priority^1,2,6,10^. A recent review by Livingston and coworkers suggests that approximately 35% of dementias is attributable to nine modifiable risk factors^2^. To prevent dementia, it is necessary to manage these risk factors including diabetes, hypertension, smoking, and obesity.

These risk factors are also related to AAA in various mechanisms and there are both commonalities and differences in the mechanisms by which the factors act on the disease^11–20^. The relationship between dementia and AAA was verified through this study, which might be due to the association of these risk factors. Although this effect cannot be completely offset, it was controlled as much as possible by analyzing through model that was adjusted using various variables including these risk factors, and there was a relationship between AAA and dementia. Since the association between these two diseases has not been revealed, we would like to propose our hypothesis through a pathophysiological approach.

The first such association is related to atherosclerosis. Traditionally, it was thought that AAA develops as a pathological response to aortic atherosclerosis. Until half a century ago, the term “atherosclerotic aneurysm” was used. This view still is favored by some researchers^21,22^. Atherosclerotic plaque growth leads to a compensatory arterial response. In other words, due to arterial narrowing, hemodynamic changes such as those in shear stress occur. The endothelium detects this and changes the phenotype of vascular smooth muscle cells, allowing remodeling through secretion of proteolytic enzymes such as metalloproteinase (MMP)^21–23^. Atherosclerosis typically is widespread throughout the vasculature, and the carotid artery is not exempt^24^. Carotid atherosclerosis and stiffness consequently cause brain microcirculation transformation and increase blood–brain barrier (BBB) permeability, leading to cognitive impairment^25^. Similarly, associations between descending thoracic aortic plaque and acceleration of brain aging have been demonstrated, and trends in accelerated progression of brain atrophy and progression of cerebrovascular lesions have been reported^26,27^.

The second association is inflammation and matrix degradation. Aortic inflammation is believed to lead to destruction of aortic media and vascular smooth muscle cell apoptosis and dysfunction. Release of a range of proteolytic enzymes, such as MMP and cysteine proteases, produces reactive oxygen, cytokines, and related products^21^. The tissue inhibitors of MMP (TIMP) are increased in the wall of the aneurysm^28^; however, the balance between TIMP and MMP seems to favor proteolysis^29^, and elastin and collagen fibers are degraded. Also, due to the paracrine effect of VSMC, a protective effect is needed to maintain homeostasis from proteolysis and inflammatory reactions^30^. However, VSMC apoptosis prevents this protective effect. These MMPs (MMP-2) induce breakdown of the BBB, disrupt oxidative homeostasis in AD^31^, and play a role in the impaired Aβ peptide metabolism responsible for progression of dementia^32^. Reactive oxygen species (ROS) affect AD by inducing oxidation of lipids, proteins, and nucleic acids and impairing Aβ clearance by the low density lipoprotein receptor-related protein (LPR1) through its oxidation^33^.

The hazard ratio of AAA for dementia in this study was 1.422, and the reason for this was deduced based on the mechanisms of AAA occurrence. However, since this deduction is only a possibility, further research is needed. In addition, we examined whether there was a difference in dementia incidence according to the treatment method of AAA, that is, the difference between the surgical group and non-surgical group or the OSAR group and EVAR group, but there were no significant differences. (Tables 3 and 4).

**Table 3:** Hazard ratio of AAA for incidence of dementia.

AAA	N	Event	Duration	Rate	Model 1	Model 2	Model 3	Model 4
**All types of dementia**								
No	45,753	3234	209,489.14	15.4376	0.666(0.621,0.713)	0.614(0.572,0.659)	0.635(0.591,0.682)	0.705(0.653,0.76)
Non-surgical	11,066	1076	46,783.84	22.9994	1	1	1	1
Surgical	4185	356	15,250.45	23.3436	1.038(0.92,1.17)	0.992(0.878,1.12)	0.982(0.869,1.109)	1.008(0.892,1.139)
**Alzheimer’s disease**								
No	45,753	2611	209,489.14	12.4637	0.709(0.656,0.767)	0.651(0.601,0.706)	0.673(0.62,0.73)	0.732(0.671,0.797)
Non-surgical	11,066	814	46,783.84	17.3992	1	1	1	1
Surgical	4185	276	15,250.45	18.0978	1.069(0.933,1.225)	1.036(0.902,1.19)	1.026(0.893,1.179)	1.047(0.911,1.203)
**Vascular dementia**								
No	45,753	332	209,489.14	1.58481	0.448(0.371,0.54)	0.427(0.352,0.518)	0.446(0.368,0.542)	0.54(0.439,0.665)
Non-surgical	11,066	165	46,783.84	3.52686	1	1	1	1
Surgical	4185	48	15,250.45	3.14745	0.902(0.654,1.244)	0.815(0.588,1.13)	0.809(0.583,1.123)	0.85(0.613,1.18)

*AAA* Abdominal aortic aneurysm, AAA group divided into surgical and non-surgical groups.

**Table 4:** Hazard ratio of AAA for incidence of dementia.

AAA	N	Event	Duration	Rate	Model 1	Model 2	Model 3	Model 4
**All types of dementia**								
No	45,753	3234	209,489.14	15.4376	0.666(0.622,0.713)	0.614(0.572,0.659)	0.635(0.591,0.682)	0.705(0.653,0.76)
Non-surgical	11,066	1076	46,783.84	22.9994	1	1	1	1
OSAR	1203	91	4859.33	18.7269	0.816(0.659,1.011)	0.929(0.749,1.152)	0.901(0.726,1.117)	0.934(0.753,1.159)
EVAR	2982	265	10,391.13	25.5025	1.144(1,1.308)	1.015(0.886,1.163)	1.013(0.884,1.161)	1.036(0.904,1.187)
**Alzheimer’s disease**								
No	45,753	2611	209,489.14	12.4637	0.709(0.656,0.767)	0.651(0.601,0.706)	0.673(0.62,0.73)	0.732(0.671,0.797)
Non-surgical	11,066	814	46,783.84	17.3992	1	1	1	1
OSAR	1203	66	4859.33	13.5821	0.783(0.609,1.006)	0.911(0.708,1.172)	0.884(0.687,1.137)	0.907(0.705,1.167)
EVAR	2982	210	10,391.13	20.2096	1.207(1.037,1.405)	1.083(0.929,1.262)	1.081(0.927,1.261)	1.101(0.944,1.284)
**Vascular dementia**								
No	45,753	332	209,489.14	1.58481	0.448(0.371,0.54)	0.427(0.352,0.518)	0.446(0.368,0.542)	0.54(0.439,0.665)
Non-surgical	11,066	165	46,783.84	3.52686	1	1	1	1
OSAR	1203	13	4859.33	2.67527	0.759(0.432,1.335)	0.78(0.442,1.375)	0.757(0.429,1.336)	0.833(0.471,1.471)
EVAR	2982	35	10,391.13	3.36826	0.97(0.673,1.397)	0.83(0.573,1.2)	0.831(0.574,1.203)	0.857(0.592,1.241)

*OSAR* Open surgical aneurysm repair, *EVAR* Endovascular aneurysm repair, Surgical group divided into OSAR and surgical groups.

Also, as documented in Supplemental Table 1, there were interactions of age, hypertension, and history of cardiovascular disease with AAA. The HR of dementia was high according to the presence or absence of AAA in the presence of hypertension, in the younger age group, and in the group with no history of cardiovascular disease. In this study, all of these factors were related to dementia, but there were differences in the directions of the interactions. Synergy was present with hypertension because it uniquely influenced dementia with AAA. However, other factors influenced the occurrence of dementia in various ways, indicating a common denominator with AAA. As a result, the HR of AAA for dementia was decreased statistically.

Based on the results revealed in this study, the relationship between AAA and Dementia should be confirmed with a prospective controlled study in the future, and it should be studied what mechanism such a relationship is caused by. In addition, although it could not be conducted in this study, it seems necessary to study whether there is a difference in the incidence of dementia in ruptured AAA and unruptured AAA patient groups.

The present study has several limitations. First, this study was a retrospective analysis. To overcome this, the influence of each variable was adjusted through a multivariate logistic regression model. However, control of the confounders among variables was not achieved. Second, diagnosis of AAA and dementia and identification of other risk factors solely were based on diagnostic codes, possibly introducing bias. For example, in the case of dementia, there is no way to confirm whether it is an accurate diagnosis made by neurologist. Third, considering the time of onset, a rough causal relationship might be inaccurately inferred because of the it is a retrospective nature of the study. Also, the degree of cognitive impairment and issues such as AAA diameter are not known, so the correlation between these could not be demonstrated clearly. Finally, due to the lack of data, the genetic factor could not be considered.

However, our study also has a number of strengths. First, we believe that this is the first study to not only demonstrate the relationship between AAA and dementia, but also to illustrate the effect of AAA on dementia in relation to each risk factor. Second, this study used a large, national sample with a relatively long follow-up period.

## Conclusions

We found that AAA was associated with increase in the risk of dementia regardless of AD or VD, even after adjusting for several comorbidities. These finding suggest that continued follow-up in AAA patients might be needed to permit early detection of signs and symptoms of dementia. In particular, surveillance is needed when high blood pressure accompanies AAA or if AAA occurs in the absence of history of heart disease in a patient younger than 65 years. However, to further confirm our findings, a large-scale prospective study is necessary in the future.

## Supplementary Information

Supplementary Information.

## References

[1] Arvanitakis Z, Shah RC, Bennett DA (2019). Diagnosis and management of dementia: Review. JAMA.

[2] Livingston G, Sommerlad A, Orgeta V (2017). Dementia prevention, intervention, and care. Lancet.

[3] Fiest KM, Jetté N, Roberts JI (2016). The prevalence and incidence of dementia: A systematic review and meta-analysis. Can. J. Neurol. Sci. J. Can. Sci. Neurol..

[4] Nordon IM, Hinchliffe RJ, Loftus IM, Thompson MM (2011). Pathophysiology and epidemiology of abdominal aortic aneurysms. Nat. Rev. Cardiol..

[5] Wang J-C, Chien W-C, Tzeng N-S (2019). Surgical repair of aortic aneurysms and reduced incidence of dementia. Int. J. Cardiol..

[6] Tisher A, Salardini A (2019). A comprehensive update on treatment of dementia. Semin. Neurol..

[7] Tublin JM, Adelstein JM, del Monte F (2019). Getting to the heart of Alzheimer disease. Circ. Res..

[8] Lee Y, Han K, Ko S-H (2016). Data analytic process of a nationwide population-based study using national health information database established by national health insurance service. Diabetes Metab. J..

[9] Wimo A, Jönsson L, Bond J (2013). The worldwide economic impact of dementia 2010. Alzheimers Dement.

[10] Perng C-H, Chang Y-C, Tzang R-F (2018). The treatment of cognitive dysfunction in dementia: A multiple treatments meta-analysis. Psychopharmacology.

[11] Biessels GJ, Despa F (2018). Cognitive decline and dementia in diabetes mellitus: Mechanisms and clinical implications. Nat. Rev. Endocrinol..

[12] Bello-Chavolla OY, Antonio-Villa NE, Vargas-Vázquez A (2019). Pathophysiological mechanisms linking type 2 diabetes and dementia: Review of evidence from clinical, translational and epidemiological research. Curr. Diabetes Rev..

[13] Walker KA, Power MC, Gottesman RF (2017). Defining the relationship between hypertension, cognitive decline, and dementia: A review. Curr. Hypertens. Rep..

[14] Roher A, Debbins M-A (2012). Cerebral blood flow in Alzheimer’s disease. Vasc. Health Risk Manag..

[15] Farruggia MC, Small DM (2019). Effects of adiposity and metabolic dysfunction on cognition: A review. Physiol. Behav..

[16] Durazzo TC, Mattsson N, Weiner MW, Alzheimer’s Disease Neuroimaging Initiative, (2014). Smoking and increased Alzheimer’s disease risk: A review of potential mechanisms. Alzheimers Dement..

[17] Raffort J, Lareyre F, Clément M (2018). Diabetes and aortic aneurysm: Current state of the art. Cardiovasc. Res..

[18] Kobeissi E, Hibino M, Pan H, Aune D (2019). Blood pressure, hypertension and the risk of abdominal aortic aneurysms: A systematic review and meta-analysis of cohort studies. Eur. J. Epidemiol..

[19] Liu C-L, Ren J, Wang Y (2020). Adipocytes promote interleukin-18 binding to its receptors during abdominal aortic aneurysm formation in mice. Eur. Heart J..

[20] Jin J, Arif B, Garcia-Fernandez F (2012). Novel mechanism of aortic aneurysm development in mice associated with smoking and leukocytes. Arterioscler Thromb. Vasc. Biol..

[21] Golledge J (2019). Abdominal aortic aneurysm: Update on pathogenesis and medical treatments. Nat. Rev. Cardiol..

[22] Golledge J, Norman PE (2010). Atherosclerosis and abdominal aortic aneurysm: Cause, response, or common risk factors?. Arterioscler Thromb. Vasc. Biol..

[23] Pasterkamp G, Strauss BH, de Kleijn D, Duckers HJ, Nabel EG, Serruys PW (2007). Arterial remodeling. Essentials of Restenosis.

[24] Yao L, Folsom AR, Alonso A (2018). Association of carotid atherosclerosis and stiffness with abdominal aortic aneurysm: The atherosclerosis risk in communities (ARIC) study. Atherosclerosis.

[25] Muhire G, Iulita MF, Vallerand D (2019). Arterial stiffness due to carotid calcification disrupts cerebral blood flow regulation and leads to cognitive deficits. J. Am. Heart Assoc..

[26] Aparicio HJ, Petrea RE, Massaro JM (2017). Association of descending thoracic aortic plaque with brain atrophy and white matter hyperintensities: The Framingham Heart Study. Atherosclerosis.

[27] van der Veen PH, Muller M, Vincken KL (2014). Longitudinal changes in brain volumes and cerebrovascular lesions on MRI in patients with manifest arterial disease: The SMART-MR study. J. Neurol. Sci..

[28] Sakalihasan N, Limet R, Defawe OD (2005). Abdominal aortic aneurysm. AAA.

[29] Tamarina NA, McMillan WD, Shively VP, Pearce WH (1997). Expression of matrix metalloproteinases and their inhibitors in aneurysms and normal aorta. Surgery.

[30] Allaire E, Muscatelli-Groux B, Mandet C (2002). Paracrine effect of vascular smooth muscle cells in the prevention of aortic aneurysm formation. J. Vasc. Surg..

[31] Wang H, Huang L, Wu L (2020). The MMP-2/TIMP-2 system in Alzheimer disease. CNS Neurol. Disord. Drug Targets.

[32] Tuna G, Yener GG, Oktay G (2018). Evaluation of matrix metalloproteinase-2 (MMP-2) and -9 (MMP-9) and their tissue Inhibitors (TIMP-1 and TIMP-2) in plasma from patients with neurodegenerative dementia. J. Alzheimers Dis..

[33] Cheignon C, Tomas M, Bonnefont-Rousselot D (2018). Oxidative stress and the amyloid beta peptide in Alzheimer’s disease. Redox. Biol..

