# Supplementary material for: The risk of dementia in adults with abdominal aortic aneurysm
Source: Sci Rep. 2022 Jan 24;12:1228. doi: 10.1038/s41598-022-05191-1 (PMC8786889; doi:10.1038/s41598-022-05191-1)
Supplement: Supplementary file 1 — Supplementary Information. [file 41598_2022_5191_MOESM1_ESM.docx]

| **Subgroup** | **AAA** | **N** | **Event** | **DURATION** | **Rate** | **Model** | **p for interaction** |
| --- | --- | --- | --- | --- | --- | --- | --- |
| **AGE_65** |  |  | **DEMENTIA** |  |  |  |  |
| Age < 65 | No | 18768 | 190 | 91640.65 | 2.0733 | 1(Ref.) | **0.0003** |
|  | Yes | 6256 | 142 | 28847.92 | 4.9224 | 1.659(1.282,2.147) |  |
| Age ≥ 65 | No | 26985 | 3044 | 117848.48 | 25.8298 | 1(Ref.) |  |
|  | Yes | 8995 | 1290 | 33186.37 | 38.8714 | 1.403(1.304,1.509) |  |
| **SEX** |  |  |  |  |  |  |  |
| Male | No | 29874 | 1820 | 135316.14 | 13.45 | 1(Ref.) | 0.5448 |
|  | Yes | 9958 | 798 | 39252.53 | 20.3299 | 1.458(1.328,1.6) |  |
| Female | No | 15879 | 1414 | 74172.99 | 19.0635 | 1(Ref.) |  |
|  | Yes | 5293 | 634 | 22781.76 | 27.8293 | 1.38(1.241,1.534) |  |
| **Diabetes** |  |  |  |  |  |  |  |
| No | No | 36694 | 2355 | 170192.27 | 13.8373 | 1(Ref.) | 0.0737 |
|  | Yes | 12055 | 1056 | 49462.31 | 21.3496 | 1.46(1.345,1.584) |  |
| Yes | No | 9059 | 879 | 39296.87 | 22.3682 | 1(Ref.) |  |
|  | Yes | 3196 | 376 | 12571.98 | 29.9078 | 1.324(1.158,1.515) |  |
| **Hypertension** |  |  |  |  |  |  |  |
| No | No | 21189 | 1095 | 99090.54 | 11.0505 | 1(Ref.) | **0.0122** |
|  | Yes | 3179 | 172 | 13962.24 | 12.3189 | 1.165(0.982,1.382) |  |
| Yes | No | 24564 | 2139 | 110398.59 | 19.3752 | 1(Ref.) |  |
|  | Yes | 12072 | 1260 | 48072.06 | 26.2107 | 1.474(1.365,1.593) |  |
| **Dyslipidemia** |  |  |  |  |  |  |  |
| No | No | 30703 | 2151 | 142729.9 | 15.0704 | 1(Ref.) | 0.3091 |
|  | Yes | 6424 | 577 | 27426.07 | 21.0384 | 1.349(1.221,1.489) |  |
| Yes | No | 15050 | 1083 | 66759.24 | 16.2225 | 1(Ref.) |  |
|  | Yes | 8827 | 855 | 34608.22 | 24.7051 | 1.503(1.361,1.66) |  |
| **CKD** |  |  |  |  |  |  |  |
| No | No | 38794 | 2383 | 178899.6 | 13.3203 | 1(Ref.) | 0.2929 |
|  | Yes | 11771 | 973 | 49338.09 | 19.7211 | 1.484(1.365,1.614) |  |
| Yes | No | 6959 | 851 | 30589.53 | 27.82 | 1(Ref.) |  |
|  | Yes | 3480 | 459 | 12696.2 | 36.1525 | 1.288(1.135,1.461) |  |
| **Cerebrovascular**  **Disease** |  |  |  |  |  |  |  |
| No | No | 43241 | 2826 | 198885.54 | 14.2092 | 1(Ref.) | 0.2778 |
|  | Yes | 12903 | 1028 | 53058.08 | 19.375 | 1.398(1.292,1.514) |  |
| Yes | No | 2512 | 408 | 10603.6 | 38.4775 | 1(Ref.) |  |
|  | Yes | 2348 | 404 | 8976.21 | 45.0079 | 1.457(1.253,1.693) |  |
| **Cardiovascular**  **Disease** |  |  |  |  |  |  |  |
| No | No | 40100 | 2612 | 185125.02 | 14.1094 | 1(Ref.) | **0.039** |
|  | Yes | 7489 | 650 | 30077.11 | 21.6111 | 1.521(1.391,1.663) |  |
| Yes | No | 5653 | 622 | 24364.12 | 25.5293 | 1(Ref.) |  |
|  | Yes | 7762 | 782 | 31957.19 | 24.4702 | 1.255(1.125,1.4) |  |

**Supplemental Table 1-1 Multivariate analysis for incidence of all types of dementia**

AAA: Abdominal aortic aneurysm, CKD: Chronic kidney disease, Rate: Incidence rate per 1000 person years, Model is adjusted for age; sex; income level; presence of diabetes, hypertension, or dyslipidemia; smoking status; alcohol consumption; exercise status; BMI; and history of CVD.

**Supplemental Table 1-2 Multivariate analysis for incidence of Alzheimer’s disease (AD)**

| **Subgroup** | **AAA** | **N** | **Event** | **DURATION** | **Rate** | **Model** | **p for interaction** |
| --- | --- | --- | --- | --- | --- | --- | --- |
| **AGE_65** |  |  | **AD** |  |  |  |  |
| Age < 65 | No | 18768 | 140 | 91640.65 | 1.5277 | 1(Ref.) | 0.0778 |
|  | Yes | 6256 | 85 | 28847.92 | 2.9465 | 1.41(1.024,1.94) |  |
| Age ≥ 65 | No | 26985 | 2471 | 117848.48 | 20.9676 | 1(Ref.) |  |
|  | Yes | 8995 | 1005 | 33186.37 | 30.2835 | 1.382(1.274,1.5) |  |
| **SEX** |  |  |  |  |  |  |  |
| Male | No | 29874 | 1449 | 135316.14 | 10.7083 | 1(Ref.) | 0.9406 |
|  | Yes | 9958 | 591 | 39252.53 | 15.0564 | 1.403(1.261,1.562) |  |
| Female | No | 15879 | 1162 | 74172.99 | 15.6661 | 1(Ref.) |  |
|  | Yes | 5293 | 499 | 22781.76 | 21.9035 | 1.355(1.204,1.525) |  |
| **Diabetes** |  |  |  |  |  |  |  |
| No | No | 36694 | 1913 | 170192.27 | 11.2402 | 1(Ref.) | 0.074 |
|  | Yes | 12055 | 812 | 49462.31 | 16.4165 | 1.43(1.304,1.568) |  |
| Yes | No | 9059 | 698 | 39296.87 | 17.7622 | 1(Ref.) |  |
|  | Yes | 3196 | 278 | 12571.98 | 22.1127 | 1.258(1.079,1.468) |  |
| **Hypertension** |  |  |  |  |  |  |  |
| No | No | 21189 | 891 | 99090.54 | 8.9918 | 1(Ref.) | **0.031** |
|  | Yes | 3179 | 134 | 13962.24 | 9.5973 | 1.158(0.954,1.404) |  |
| Yes | No | 24564 | 1720 | 110398.59 | 15.5799 | 1(Ref.) |  |
|  | Yes | 12072 | 956 | 48072.06 | 19.8868 | 1.426(1.306,1.556) |  |
| **Dyslipidemia** |  |  |  |  |  |  |  |
| No | No | 30703 | 1725 | 142729.9 | 12.0858 | 1(Ref.) | 0.7799 |
|  | Yes | 6424 | 447 | 27426.07 | 16.2984 | 1.352(1.209,1.513) |  |
| Yes | No | 15050 | 886 | 66759.24 | 13.2716 | 1(Ref.) |  |
|  | Yes | 8827 | 643 | 34608.22 | 18.5794 | 1.418(1.267,1.587) |  |
| **CKD** |  |  |  |  |  |  |  |
| No | No | 38794 | 1924 | 178899.6 | 10.7546 | 1(Ref.) | 0.3995 |
|  | Yes | 11771 | 740 | 49338.09 | 14.9986 | 1.44(1.31,1.584) |  |
| Yes | No | 6959 | 687 | 30589.53 | 22.4587 | 1(Ref.) |  |
|  | Yes | 3480 | 350 | 12696.2 | 27.5673 | 1.259(1.091,1.452) |  |
| **Cerebrovascular**  **Disease** |  |  |  |  |  |  |  |
| No | No | 43241 | 2321 | 198885.54 | 11.67 | 1(Ref.) | 0.1848 |
|  | Yes | 12903 | 807 | 53058.08 | 15.2097 | 1.357(1.242,1.483) |  |
| Yes | No | 2512 | 290 | 10603.6 | 27.3492 | 1(Ref.) |  |
|  | Yes | 2348 | 283 | 8976.21 | 31.5278 | 1.45(1.212,1.734) |  |
| **Cardiovascular**  **Disease** |  |  |  |  |  |  |  |
| No | No | 40100 | 2111 | 185125.02 | 11.4031 | 1(Ref.) | **0.0256** |
|  | Yes | 7489 | 504 | 30077.11 | 16.7569 | 1.499(1.355,1.658) |  |
| Yes | No | 5653 | 500 | 24364.12 | 20.522 | 1(Ref.) |  |
|  | Yes | 7762 | 586 | 31957.19 | 18.337 | 1.196(1.057,1.354) |  |

AAA: Abdominal aortic aneurysm, CKD: Chronic kidney disease, Rate: Incidence rate per 1000 person years, Model is adjusted for age; sex; income level; presence of diabetes, hypertension, or dyslipidemia; smoking status; alcohol consumption; exercise status; BMI; and history of CVD.

**Supplemental Table 1-3 Multivariate analysis for incidence of vascular dementia (VD)**

| **Subgroup** | **AAA** | **N** | **Event** | **DURATION** | **Rate** | **Model** | **p for interaction** |
| --- | --- | --- | --- | --- | --- | --- | --- |
| **AGE_65** |  |  | **VD** |  |  |  |  |
| Age < 65 | No | 18768 | 31 | 91640.65 | 0.33828 | 1(Ref.) | **0.0058** |
|  | Yes | 6256 | 42 | 28847.92 | 1.45591 | 2.351(1.364,4.05) |  |
| Age ≥ 65 | No | 26985 | 301 | 117848.48 | 2.55413 | 1(Ref.) |  |
|  | Yes | 8995 | 171 | 33186.37 | 5.15272 | 1.681(1.36,2.078) |  |
| **SEX** |  |  |  |  |  |  |  |
| Male | No | 29874 | 200 | 135316.14 | 1.47802 | 1(Ref.) | 0.5799 |
|  | Yes | 9958 | 129 | 39252.53 | 3.28641 | 1.814(1.41,2.335) |  |
| Female | No | 15879 | 132 | 74172.99 | 1.77962 | 1(Ref.) |  |
|  | Yes | 5293 | 84 | 22781.76 | 3.68716 | 1.765(1.292,2.411) |  |
| **Diabetes** |  |  |  |  |  |  |  |
| No | No | 36694 | 227 | 170192.27 | 1.33379 | 1(Ref.) | 0.3071 |
|  | Yes | 12055 | 151 | 49462.31 | 3.05283 | 1.837(1.454,2.322) |  |
| Yes | No | 9059 | 105 | 39296.87 | 2.67197 | 1(Ref.) |  |
|  | Yes | 3196 | 62 | 12571.98 | 4.9316 | 1.679(1.176,2.398) |  |
| **Hypertension** |  |  |  |  |  |  |  |
| No | No | 21189 | 102 | 99090.54 | 1.02936 | 1(Ref.) | 0.1071 |
|  | Yes | 3179 | 18 | 13962.24 | 1.28919 | 1.12(0.654,1.919) |  |
| Yes | No | 24564 | 230 | 110398.59 | 2.08336 | 1(Ref.) |  |
|  | Yes | 12072 | 195 | 48072.06 | 4.05641 | 1.918(1.549,2.373) |  |
| **Dyslipidemia** |  |  |  |  |  |  |  |
| No | No | 30703 | 231 | 142729.9 | 1.61844 | 1(Ref.) | 0.1668 |
|  | Yes | 6424 | 84 | 27426.07 | 3.06278 | 1.523(1.159,2.002) |  |
| Yes | No | 15050 | 101 | 66759.24 | 1.5129 | 1(Ref.) |  |
|  | Yes | 8827 | 129 | 34608.22 | 3.72744 | 2.144(1.606,2.862) |  |
| **CKD** |  |  |  |  |  |  |  |
| No | No | 38794 | 247 | 178899.6 | 1.38066 | 1(Ref.) | 0.8562 |
|  | Yes | 11771 | 145 | 49338.09 | 2.93891 | 1.823(1.443,2.303) |  |
| Yes | No | 6959 | 85 | 30589.53 | 2.77873 | 1(Ref.) |  |
|  | Yes | 3480 | 68 | 12696.2 | 5.35593 | 1.663(1.162,2.38) |  |
| **Cerebrovascular**  **disease** |  |  |  |  |  |  |  |
| No | No | 43241 | 259 | 198885.54 | 1.30226 | 1(Ref.) | 0.563 |
|  | Yes | 12903 | 129 | 53058.08 | 2.4313 | 1.798(1.419,2.278) |  |
| Yes | No | 2512 | 73 | 10603.6 | 6.88446 | 1(Ref.) |  |
|  | Yes | 2348 | 84 | 8976.21 | 9.35807 | 1.631(1.157,2.298) |  |
| **Cardiovascular**  **disease** |  |  |  |  |  |  |  |
| No | No | 40100 | 268 | 185125.02 | 1.44767 | 1(Ref.) | 0.6184 |
|  | Yes | 7489 | 95 | 30077.11 | 3.15855 | 1.862(1.455,2.381) |  |
| Yes | No | 5653 | 64 | 24364.12 | 2.62681 | 1(Ref.) |  |
|  | Yes | 7762 | 118 | 31957.19 | 3.69244 | 1.632(1.189,2.24) |  |

AAA: Abdominal aortic aneurysm, CKD: Chronic kidney disease, Rate: Incidence rate per 1000 person years, Model is adjusted for age; sex; income level; presence of diabetes, hypertension, or dyslipidemia; smoking status; alcohol consumption; exercise status; BMI; and history of CVD.
